# Soybean-Hop Alleviates Estrogen Deficiency-Related Bone Loss and Metabolic Dysfunction in Ovariectomized Rats Fed a High-Fat Diet

**DOI:** 10.3390/molecules23051205

**Published:** 2018-05-17

**Authors:** Dasom Noh, Yeni Lim, Hansol Lee, Hyejin Kim, Oran Kwon

**Affiliations:** 1 Department of Nutritional Science and Food Management, Ewha Womans University, Seoul 03760, Korea; nds8195@naver.com (D.N.); ynlim@ewha.ac.kr (Y.L.); 1ee9930812@gmail.com (H.L.); 2 Department of Kinesiology and Sports Studies, Ewha Womans University, Seoul 03760, Korea; shine1215@hotmail.com

**Keywords:** soybeans, hops, ovariectomized rats, bone loss, metabolic dysfunction

## Abstract

Soybeans and hops have been traditionally used as a natural estrogen replacement therapy and their major active ingredients, isoflavones and prenylflavanones, are known to have estrogenic/antiestrogenic effects depending on the target organ. However, their potential benefits are still subject to controversies. The present study investigated the dual effect of soy isoflavones plus hop prenylflavanones (Soy-Hop) on bone loss and metabolic dysfunction under estrogen deficient condition. Rats were sham-operated (*n* = 10) or ovariectomized (OVX; *n* = 40) and then fed a high-fat diet (HFD) to develop hyperlipidemia in OVX rats within the experimental period of 8 weeks. The OVX/HFD rats were assigned to four groups to receive different doses of Soy-Hop (0, 30, 100, and 300 mg/kg) by oral gavage for 8 weeks. High-dose Soy-Hop significantly suppressed OVX/HFD-induced increases in food intake, body weight gain, fat mass, and circulating levels of leptin, adiponectin, LDL-cholesterol, total cholesterol, triglycerides, glucose, and insulin. High-dose Soy-Hop also attenuated OVX/HFD-induced elevation of osteocalcin, alkaline phosphatase, and CTX in plasma and RANKL/OPG gene expression ratio in femur. These findings were confirmed visually by confocal analysis of GLUT4 translocation in soleus muscle cells and micro-computed tomography scanning of the distal femoral epiphysis, respectively. These results suggest that Soy-Hop may have potential to ameliorate estrogen deficiency-related alterations in both metabolism and bone quality, at least in part, by hormonal factors secreted by adipocytes.

## 1. Introduction

Estrogen is known to have key roles regulating diverse functions in human physiology, including reproductive function, glucose and lipid metabolism, bone metabolism, and neurological functions [1]. Therefore, the postmenopausal decline in circulating estrogen levels are related to the occurrence of multiple physiological defects, leading to the development of cardiovascular disease, type II diabetes, and osteoporosis [2,3,4]. Given that addition of estrogen may prevent or treat many physiological alterations that occur with or following the menopausal transition, hormone therapy (HT) has been used for 80 years. However, the use of HT has declined sharply due to increased concerns about adverse events and cancer development after the release of the Women’s Health Initiative findings in 2002 [5,6]. Instead, the use of complementary and alternative approaches has increased for symptom relief and improved quality of life. Dietary intervention is one of the important fields that has received increasing attention.

Historically, soybeans and hops have been used as a natural estrogen replacement therapy. Currently, a significant body of scientific evidence has been advanced to support the estrogenic effects of isoflavones (genistein, daidzein, glycitein, daidzin, genistin, and glycitin), mostly isolated from soybeans [7,8]. Although not recognized as such, prenylflavanones (8-prenylnaringenin, 6-prenylnaringenin, isoxanthohumol, and xanthohumol) isolated from hops are also receiving increasing attention as an alternative approach for HT with the reliable results of experiments [9,10,11]. Furthermore, in vitro and in vivo (animal) studies suggested that isoflavones and prenylflavanones exert both estrogenic and antiestrogenic effects depending on the target tissues [12,13]. They act as antagonists that weaken the estrogenic action on uterine and breast tissue, while exerting estrogenic effects on bone and blood vessels. This potential of organ selectivity may be viewed as a great benefit of using phytoestrogens [12]. 

However, potential benefits of soy isoflavones and hop prenylflavanones are still subject to controversies. In a recent review, Keiler et al. [11] stated that soy isoflavones are not a sufficient alternative to HT since their effectiveness is dependent on lifelong exposure. Instead, they indicated that hop prenylflavones seem to be more promising. Moreover, there are not yet any studies on the effects of a combination of soy isoflavones and hop prenylflavanones (Soy-Hop) against estrogen deficiency-induced abnormalities in metabolism. Therefore, we performed this study to investigate whether Soy-Hop could ameliorate bone loss and metabolic dysfunction found in an ovariectomized (OVX)/high-fat diet (HFD) rat model. HFD feeding was employed in OVX rats to develop hyperlipidemia in a relatively short period, mimicking postmenopausal cardiovascular disease [14,15,16]. Furthermore, to explore the underlying mechanisms involved in its effects, we determined various biochemical parameters of lipid and bone metabolism in plasma, receptor-activator of nuclear factor kB ligand (RANKL), and osteoprotegerin (OPG) gene expressions in femur, confocal analysis of glucose transporter 4 (GLUT4) translocation in soleus muscle cells, and microcomputed tomography (micro-CT) scanning of the distal femoral epiphysis.

## 2. Results

### 2.1. Effects of Soy-Hop on Food Intake, Body Weight, and Metabolic Parameters

Compared to the sham group, OVX control group showed significant increases in food intake, body weight gain, and fat pad weight and a decrease in uterus weight. However, Soy-Hop significantly decreased these parameters dose-dependently and the most significant reduction was found at a high-dose, almost to the sham level. In contrast, uterus weight was not changed by Soy-Hop administration in OVX/HFD rats. No significant difference was found in weights of muscle and femur among all groups. Consistent with the results of fat pad weights, circulating leptin, adiponectin, and low-density lipoprotein (LDL)-cholesterol levels were significantly increased by OVX, but reduced by Soy-Hop dose-dependently showing notable suppression in high-dose group. The same trend was found in total cholesterol (TC), triglycerides (TG), fasting blood glucose (FBG), insulin, C-peptide, and homeostasis model assessment-insulin resistance (HOMA-IR) levels, resulting in similar or even lower values in high-dose group than those in sham group. The high-density lipoprotein (HDL)-cholesterol and estradiol levels were not changed in all groups (Table 1).

**Figure 1:**
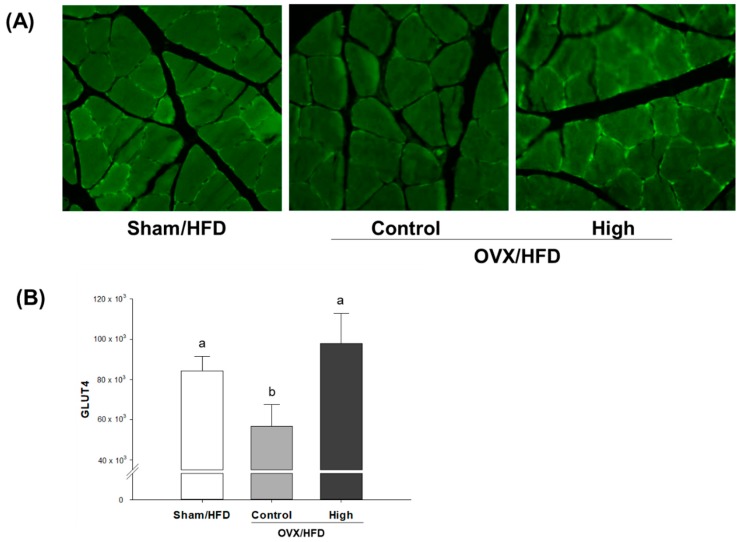
Confocal immunohistochemical analysis of (**A**) soleus muscle GLUT4 protein and (**B**) its quantification in OVX/HFD rats. Rats were orally administered with saline or 300 mg/kg of Soy-Hop for 8 weeks, showing increased GLUT4 translocation to the membrane surface by Soy-Hop. Sham, sham-operation; OVX, ovariectomized; HFD, high-fat diet; High, 300 mg/kg Soy-Hop; GLUT4, glucose transporter 4. Values are presented as the means ± SEM (*n* = 10 per group). Bars with different letters are significantly different at *p* < 0.05 as analyzed by Duncan’s multiple comparison test.

For visualization of the effect of high-dose Soy-Hop on OVX-induced metabolic dysfunction, we determined GLUT4 translocation in soleus muscle cells using an endofacial GLUT4 antibody (Figure 1). There is little translocation of GLUT4 protein from intracellular compartments to the cell surface in OVX control group. However, Soy-Hop induced significant translocation of GLUT4 protein, as indicated by small bright green spots in cell membrane. The translocation was even better than that found in sham group.

### 2.2. Effects of Soy-Hop on Dynamics of Bone Formation and Bone Resorption

Bone formation was assessed by measuring osteocalcin and alkaline phosphatase (ALP) levels in plasma; and bone resorption was assessed by collagen type 1 cross-linked C telopeptide (CTX) and collagen type 1 cross-linked N telopeptide (NTX) levels in plasma. The OVX-induced elevation of osteocalcin, ALP, and CTX levels were significantly decreased by Soy-Hop administration in a dose-dependent manner. The same pattern was observed in NTX level, although not statistically significant. Then, the mRNA expressions for osteoclastic (RANKL) and osteogenic (OPG) activities were determined in femur by real-time polymerase chain reaction (RT-PCR). Soy-Hop significantly attenuated the OVX-induced elevation in RANKL mRNA expression in a dose-dependent manner, while no statistically significant alteration was found in OPG mRNA expression. Accordingly, these changes brought a positive effect on bone remodeling, which is mainly controlled in terms of RANKL/OPG ratio (Table 2).

**Figure 2:**
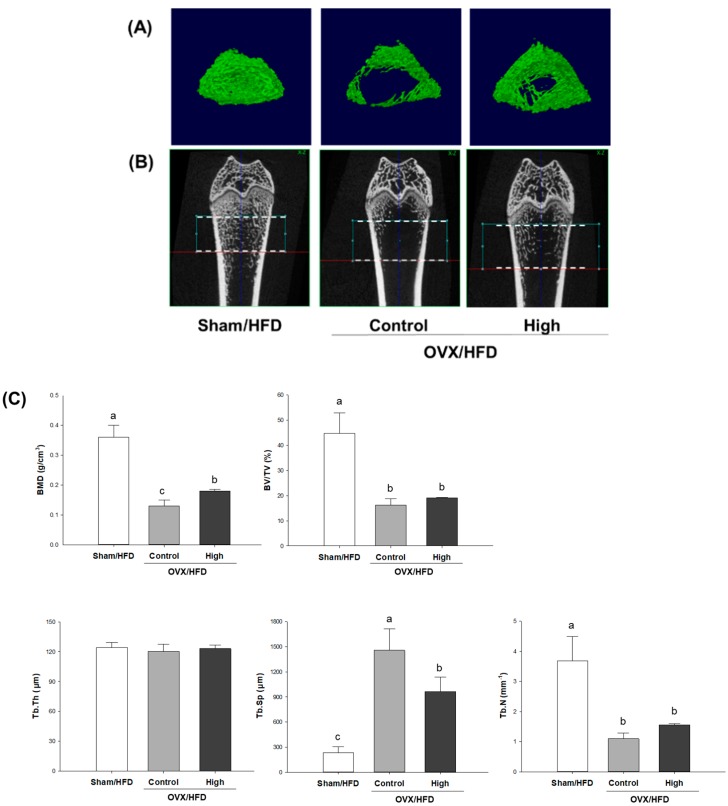
Micro-computed tomography analysis of distal femoral epiphysis in (**A**) two-dimensional plane, (**B**) three-dimensional reconstruction, and (**C**) its quantification in OVX/HFD rats. Rats were orally administered with saline or 300 mg/kg of Soy-Hop for 8 weeks, showing noticeable changes in the porosity of the bone microarchitecture and decreased trabecular separation by Soy-Hop, Sham, sham-operation; OVX, ovariectomized; HFD, high-fat diet; High, 300 mg/kg Soy-Hop; BMD, bone mineral density; BV/TV, bone volume percent; Tb.Th, trabecular thickness; Tb.Sp, trabecular space; Tb.N, trabecular number. Values are presented as the means ± SEM (*n* = 10 per group). Bars with different letters are significantly different at *p* < 0.05 as analyzed by Duncan’s multiple comparison test.

For visualization of the effect of high-dose Soy-Hop on morphologic changes of bones, we analyzed micro-CT images of distal femoral epiphysis (Figure 2). Significant deterioration of the trabecular microarchitecture was observed in OVX control group compared to that in sham group. The mean values of bone mineral density, bone volume percent, trabecular space, and trabecular number were significantly changed in OVX control group compared with that of sham group. No difference was found for the trabecular thickness. However, Soy-Hop noticeably reduced the porosity of the microarchitecture and decreased the separation between trabeculae. In particular, bone mineral density and trabecular space were significantly altered by Soy-Hop administration, although not reaching to the level seen in sham group.

## 3. Discussion

In the present study, we observed that Soy-Hop, a combination of soy isoflavones and hop prenylflavanones, exerted estrogen-like effects on bone loss and metabolic dysfunction found in OVX/HFD rats. The dose of isoflavones tested in this study ranged from 3.3 to 33 mg/kg body weight/day as sum of daidzein, genistein, glycitein, daidzin, genistin, and glycitin; and the dose of prenylflavanones was ranged from 0.029 to 0.29 mg/kg body weight/day as 8-prenylnaringenin. The tested doses of isoflavones plus prenylflavanones could be considered safe because any signs of toxicity such as uterine hypertrophy were not found. The practical application of the major findings of this study is that Soy-Hop can improve quality of life in individuals with estrogen deficiency through bone-sparing and metabolism-ameliorating effects. 

Availability of a reliable animal model would be highly beneficial to understanding the molecular pathways involved in its effect. Withdrawal of estrogen after bilateral OVX in rodents is a scientifically accepted model for investigating problems related to the postmenopausal condition [14,17]. However, in fact, it takes too long to develop hyperlipidemia in OVX rodents. Thus, HFD feeding was often employed in OVX rodents to study postmenopausal cardiovascular disease (CVD) in OVX rodents [14,15,16]. In the present study, we also adopted this strategy for studying the dual role of Soy-Hop in preventing estrogen deficiency-related bone loss as well as dyslipidemia. The results of the present study added another evidence to confirm that OVX rats fed with HFD for 8 weeks aggravated the effect of OVX, leading to metabolic changes including increased fat pad accumulation and dyslipidemia. 

Our first finding on the role of Soy-Hop in OVX/HFD rats is that high-dose Soy-Hop significantly reduced food intake, body weight gain, fat pad accumulation, and lipid/glucose levels in fasting blood. Obesity and abnormal lipid/glucose profile are common in postmenopausal women and those with CVD, and thus often used as a target for development of phytoestrogenic agents. Then, to gain insight into the mechanism how Soy-Hop exerts its effect against OVX/HFD-induced metabolic alterations, we measured leptin and adiponectin levels in plasma and visualized insulin-dependent GLUT4 translocation to cell membrane in soleus muscle cells. Our result revealed that OVX/HFD rats had the highest concentration of plasma leptin and adiponectin. This is in line with results of Hong et al. [18], who suggested that the increase in obesity caused an increase in leptin in postmenopausal women, significantly contributing to insulin resistance. Although the mechanisms involved in regulating adiponectin are not clear, some studies demonstrated that postmenopausal women also had the highest adiponectin levels compared with premenopausal women [19]. Part of it was explained by the fact that menopause transition might be associated with an increase in body fat mass [20] and a decrease in adiponectin clearance in the kidney [21]. However, it appeared that changes of adiponectin in postmenopausal women were independently related to leptin and insulin resistance values [19]. In this study, following oral administration of Soy-Hop, significantly lower levels of circulating leptin and adiponectin were found in OVX/HFD rats. In addition, we found that Soy-Hop played an important role in stimulating GLUT4 translocation to the plasma membrane as assessed by confocal image analysis. GLUT4 is an insulin-responsive glucose transporter that plays an important role in whole-body glucose clearance under physiological conditions [22].

Next, we found that oral administration of Soy-Hop significantly attenuated OVX-induced bone loss. The trabecular bone is thin and has a larger surface area, so that it is known to be metabolically active and more responsive to dietary interventions as compared with the cortical bone [23]. In the present study, micro-CT scanning of the trabecular femur revealed that OVX-induced microarchitectural deteriorations were visibly improved by high-dose Soy-Hop. Especially two trabecular microstructural parameters, density and separation, were significantly preserved by Soy-Hop administration, although not to the level of the sham-control level due to the relatively short time period. Furthermore, the dynamics of the metabolic balance between bone formation and resorption was determined. Bone formation was assessed by measuring plasma ALP and osteocalcin. ALP is a membrane-bound enzyme that plays an important role in osteoid formation and mineralization [24]. Osteocalcin is a specific marker for osteoblast function, as it is a hydroxyapatite-binding protein exclusively produced by osteoblasts [25]. Bone resorption was assessed by measuring plasma NTX and CTX, which are collagen fragments released during bone remodeling [26]. CTX was more responsive to SH supplementation than NTX. In addition, we determined the mRNA expression of two major cytokines, OPG and RANKL, which regulate osteoclast differentiation and activation [27]. RANKL is released from osteoblasts and activates RANK in osteoclasts, triggering osteoclast maturation and bone resorption [28]. OPG is also produced by osteoblasts, but acts as a decoy receptor by blocking RANKL binding to RANK. Thus, the balance of the expression of RANKL (stimulator) and OPG (inhibitor) is important in maintaining bone homeostasis [29]. In the current study, Soy-Hop significantly reduced the OVX/HFD-induced increase in RANKL/OPG ratio. Taken together, the data obtained in this study indicate that oral administration of Soy-Hop also improved bone quality by suppressing the OVX-induced increase in bone turnover. Measurement of bone turnover markers has been reported to provide a better basis than BMD for evaluating early response in preventing or reducing the risk of osteoporosis [26].

Osteoporosis and CVD are the most prevalent diseases in menopause. Accumulating evidence indicates that they appear to have pathophysiologic interactions [30]. In the present study, to the best our knowledge, we have for the first time demonstrated that Soy-Hop has dual potential for modifying the risks of osteoporosis as well as CVD in postmenopausal women. In addition, our data showed that plasma leptin has a negative correlation with bone mineral density, and a positive correlation with body weight increase, fat pad accumulation, and plasma lipids. Some clinical studies support this finding by reporting a negative association between leptin and BMD in post-menopausal women [31,32]. Chedraui et al. [33] reported that postmenopausal women with the metabolic syndrome displayed leptin resistance. Taken together, although further validation is still needed, the results of the present study suggest that the protective effect of Soy-Hop on OVX-induced alterations in both metabolism and bone quality might be mediated, at least in part, by hormonal factors secreted by adipocytes. 

## 4. Materials and Methods

### 4.1. Materials

**Figure 3:**
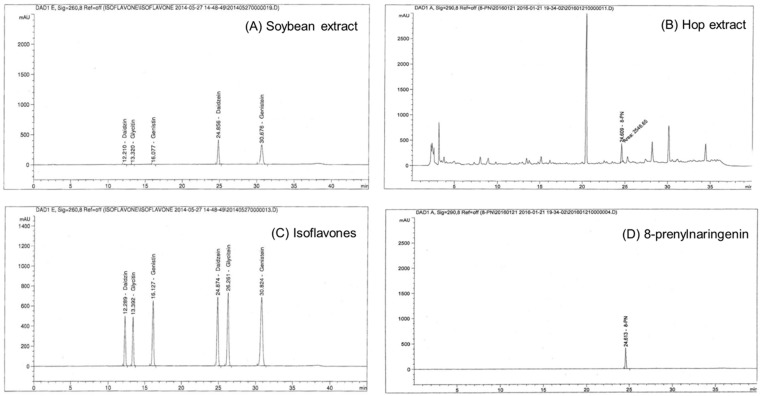
High-performance liquid chromatogram of (**A**) soybean extract, (**B**) hop extract, (**C**) isoflavones (daidzein, genistein, glycitein, daidzin, genistin, and glycitin) standard, and (**D**) 8-prenylnaringenin standard. Analytical conditions are described in materials.

The Soy-Hop was kindly provided by Pulmuone Co., Ltd. (Seoul, Korea). Briefly, soybeans (*Glycine max* L.) were extracted with ethanol at 60–70 °C for 2 h, concentrated under reduced pressure and then spray-dried. Hops (*Humulus lupulus* L.) were extracted with ethanol supercritical carbon dioxide, followed by concentration and spray-drying. Each powder was mixed at a ratio of 7:12 to form a test material. Isoflavones and prenylflavanones were analyzed using a high-performance liquid chromatography system equipped with a UV detector (Agilent technologies, Santa Clara, CA, USA) and a Shiseido Capcell Pak C18 column (250 × 4.6 mm, 5 µm; Shiseido, Tokyo, Japan). For isoflavones, the mobile phase was changed from 90% A for 0–21 min, 60% A for 21–35 min, 40% A for 35–36 min, and 90% A for 36–45 min, where solvent A is 2% acetic acid in 10% methanol and solvent B is 2% acetic acid in methanol. For prenylflavanones, the mobile phase was changed from 75% A for 4–30 min, 20% A for 30–33 min, and 100% B for 35–45 min, where solvent A is water and solvent B is 0.1% formic acid in acetonitrile. Soybean extract was standardized with isoflavones (sum of daidzein, genistein, glycitein, daidzin, genistin, and glycitin) at a concentration of 300–350 mg/g, and hop extract was standardized with 8-prenylnaringenin at a concentration of 1.5–2.5 mg/g (Figure 3).

### 4.2. Animals and Treatments

Eight-week-old female Sprague-Dawley rats were purchased from Central Lab Animal Inc. (Seoul, Korea). The rats were housed individually with a 12 h light/dark cycle at a temperature of 23 ± 1 °C and a humidity level of 45 ± 5%. After one week of acclimatization, rats were subjected to a bilateral OVX or sham surgery under anesthesia and subsequently maintained on an HFD during the whole experimental period (Feedlab; Guri, Korea). The HFD provided 45% kcal fat, 35% kcal carbohydrate and 20% kcal protein (Table 3). One week after the operation, rats were randomized into four groups to receive 30 mg/kg (Low), 100 mg/kg (Medium), or 300 mg/kg (High) of Soy-Hop or its vehicle (saline, Control) by oral gavage for 8 weeks (*n* = 10/group). The sham group was also maintained on an HFD and treated with the vehicle. Food intake and body weight were measured twice a week, and the data were summarized as total food intake and total body weight gain over the experimental period. 

At the end of the experimental period, overnight-fasted animals were euthanized by carbon dioxide, and blood samples were collected in an ethylene diamine tetra acetic acid tube by cardiac puncture. The blood samples were centrifuged (3000× *g*, 4 °C, 30 min) to obtain plasma, and then kept frozen at −70 °C until analysis. The fat pads (perirenal, retroperitoneal, and cervical), right femur bones, soleus muscles, and uterus were removed, weighed, and snap-frozen in a freezer at −70 °C until use. Alternatively, bones and muscles were fixed and preserved in 4% formalin solution (Sigma, St. Louis, MO, USA) for histological analysis. The experimental protocol was approved by the Institutional Animal Care and Use Committee of Ewha Womans University (Seoul, Korea, Approval Number: 16-004), and all experimental procedures were conducted in compliance with the National Research Council’s Guide for the Care and Use of Laboratory Animals.

### 4.3. Biochemical Assays

Plasma TC, HDL-cholesterol, and TG levels were analyzed using commercial enzymatic kits (Asan Pharmaceutical, Seoul, Korea). LDL-cholesterol was calculated by the Friedewald’s formula: LDL-cholesterol = TC − HDL-cholesterol − TG/5. Glucose oxidase-based kits (Asan Pharmaceutical) were used to determine FBG levels according to the manufacturer’s protocol. Enzyme-linked immunosorbent assay (ELISA) kits were used to determine leptin and adiponectin (R&D Systems, Minneapolis, MN, USA), insulin and C-peptide (Mercodia, Uppsala, Sweden), osteocalcin, ALP, CTX, and NTX (Cusabio Biotech, Wuhan, China), and estradiol (Biovision, Milpitas, CA, USA) levels in plasma according to the manufacturer’s protocol. HOMA-IR was calculated using the following equation [34]: HOMA-IR = Fasting plasma insulin (µU/mL) × Fasting blood glucose (mmol/L)/22.5.

### 4.4. Quantitative Real-Time PCR Analysis

Total RNA was extracted from femurs using TRIzol reagent (Ambion, Austin, TX, USA). RNA concentration and quality were measured at 260/280 using BioSpec-nano (Shimadzu Corp., Tokyo, Japan) and converted into cDNA using a high-capacity cDNA reverse transcription kit (Applied Biosystems, Foster City, CA, USA). cDNA was mixed with SYBR Green master mix (Kapa Biosystems, Wilmington, MA, USA) and analyzed by a step-one-plus RT-PCR system (Applied Biosystems). The gene-specific primers were used for RANKL, OPG, and β-actin are listed in Table 4. Primers sequenced at Macrogen (Seoul, Korea) and compared with sequences deposited at the National Center for Biotechnology Information (NCBI) using BLAST alignment programs (http://www.ncbi.nlm.nih.gov/BLAST/). Amplifications were performed starting with a 3-min melting step at 95 °C, followed by 40 cycles of 95 °C for 3 s and 60 °C for 30 s. The conditions of the final melting curve stage were as follows: 95 °C for 15 s, 60 °C for 1 min, and 95 °C for 15 s. The relative amounts of all of the RNAs were normalized to the amount of β-actin, and the relative amounts of the RNAs were calculated using the 2^−ΔΔ*C*t^ method described by K. Livak et al. [35]. Values were expressed as a fold change over the Sham/HFD group.

### 4.5. Immunohistochemical Staining

Soleus muscles were fixed in 10% neutral buffered formalin solution. Cross sections were cut from the middle region of each muscle. Paraffin sections were deparaffinized, hydrated, and subjected to antigen retrieval by xylene. The tissue was placed in 0.02% Triton X-100 in phosphate-buffered saline (PBST) for 15 min (permeabilization) and moved into 5% bovine serum albumin in PBST for 30 min (blocking). Next, the samples were washed with phosphate-buffered saline (PBS) and probed with a GLUT) polyclonal rabbit antibody (Abcam, Cambridge, UK) in 3% BSA in PBS and incubated overnight at 4 °C. The slide washing step was repeated three times for 5 min in 0.05% Tween 20 in PBS. The samples were incubated with Alexa 488-conjugated goat anti-rabbit IgG secondary antibody (Invitrogen, Carlsbad, CA, USA) in PBS that contained 3% BSA for 20 min at room temperature. After washing three times with 0.05% Tween 20 in PBS, mounting media was put on the slides (Vector Laboratories, Burlingame, CA, USA). The protein levels of GLUT4 in the membrane surface and cytosol were imaged using an LSM-510 Meta confocal microscope (Carl Zeiss, Oberkochen, Germany).

### 4.6. Microcomputed Tomography (Micro-CT) Scanning

The right femur was fixed with 10% neutral buffered formalin solution (Sigma, St. Louis, MO, USA). For morphologic assessments of the distal femoral epiphysis, block samples were scanned using SkyScan1176 micro-CT scanner (Bruker, Kontich, Belgium) employing a 50 kV/200 µA tungsten X-ray source and a 5.0 mm aluminum filter.

### 4.7. Statistical Analyses

The results are expressed as the means ± SEM. Statistical analyses were performed using SAS 9.4 (SAS institute Inc., Cary, NC, USA). Normal distribution was confirmed by Kolmogorov–Smirnov tests. The differences among all the groups were analyzed by one-way analysis of variance (ANOVA) with the post hoc Duncan’s multiple comparison test. Statistical significance was considered at *p* < 0.05.

## 5. Conclusions

Our findings suggest the following: (1) Soy-Hop alleviated estrogen deficiency-related bone loss by suppressing bone turnover; (2) Soy-Hop ameliorated estrogen deficiency-related metabolic dysfunction by stimulating leptin and insulin sensitivity; (3) The protective effect of Soy-Hop on OVX-induced alterations in both metabolism and bone quality might be mediated, at least in part, by hormonal factors secreted by adipocytes. One of the limitations of this study is that the experiment was designed in the absence of normal diet group, so that one can say that the causality of the results is difficult to interpret. However, our main purpose here was not to focus on the effect of HFD in OVX rats, but to evaluate the dual effect of Soy-Hop on bone and lipid metabolism under estrogen deficient conditions. A second limitation is that we did not determine the effects of individual signature components, isoflavones and prenylflavanones, which might explain the synergistic effects of the combination of Soy and Hop in protection of estrogen-deficiency related metabolic dysfunctions. This will be the focus of future research.

## Figures and Tables

**Table 1:** Effect of Soy-Hop on food intakes, body weight changes, and metabolic parameters in OVX/HFD rats. Rats were orally administered with Soy-Hop (30, 100, and 300 mg/kg) for 8 weeks.

Variables	Sham/HFD	OVX/HFD				*p*-Values
		Control	Low	Medium	High	
Total food intake (g)	520.2 ± 15.9 ^bc^	587.3 ± 18.0 ^a^	538.5 ± 12.7 ^bc^	554.9 ± 16.3 ^ab^	502.0 ± 14.9 ^c^	0.0045
Total body weight gain (g)	118.1 ± 11.1 ^cd^	181.0 ± 12.5 ^a^	146.6 ± 7.5 ^bc^	151.2 ± 8.6 ^b^	110.0 ± 9.7 ^d^	<0.0001
Total fat pad (g)	30.4 ± 3.3 ^b^	41.3 ± 3.2 ^a^	34.6 ± 2.1 ^ab^	31.7 ± 2.3 ^b^	27.6 ± 2.1 ^b^	0.0081
Soleus muscle weight (mg)	236.0 ± 12.5	266.0 ± 12.7	260.0 ± 8.4	262.0 ± 7.9	238.0 ± 8.8	0.1226
Femur weight (mg)	905.5 ± 16.3	882.0 ± 22.7	878.5 ± 14.9	865.0 ± 19.7	880.0 ± 24.6	0.4770
Uterus weight (mg)	514.0 ± 35.2 ^a^	122.0 ± 8.4 ^b^	138.0 ± 24.4 ^b^	101.0 ± 9.8 ^b^	151.0 ± 21.2 ^b^	<0.0001
Leptin (ng/mL)	9.3 ± 1.7 ^b^	17.9 ± 2.2 ^a^	14.2 ± 2.5 ^ab^	10.9 ± 1.5 ^b^	9.3 ± 1.5 ^b^	0.0106
Adiponectin (μg/mL)	7.3 ± 0.3 ^c^	11.7 ± 0.8 ^a^	10.7 ± 1.3 ^ab^	12.3 ± 0.8 ^a^	8.8 ± 0.5 ^bc^	0.0001
LDL-cholesterol (mg/dL)	29.3 ± 3.9 ^c^	62.4 ± 9.3 ^a^	47.9 ± 6.9 ^abc^	50.9 ± 5.3 ^ab^	31.4 ± 6.1 ^bc^	0.0034
HDL-cholesterol (mg/dL)	61.5 ± 6.2	58.4 ± 6.4	62.0 ± 8.2	47.3 ± 2.1	44.8 ± 5.0	0.1178
TC (mg/dL)	113.0 ± 6.3 ^ab^	139.5 ± 13.0 ^a^	129.6 ± 9.0 ^a^	113.2 ± 6.7 ^ab^	88.9 ± 8.5 ^b^	0.0035
TG (mg/dL)	96.6 ± 9.8 ^a^	93.7 ± 13.0 ^a^	86.3 ± 7.6 ^a^	74.9 ± 6.0 ^ab^	59.1 ± 3.0 ^b^	0.0283
FBG (mmol/L)	15.8 ± 0.9 ^c^	22.2 ± 1.4 ^a^	19.6 ± 1.5 ^ab^	19.2 ± 1.1 ^abc^	17.2 ± 1.0 ^bc^	0.0050
Insulin (μg/L)	34.4 ± 4.7 ^ab^	34.9 ± 3.1 ^ab^	46.7 ± 6.8 ^a^	31.3 ± 4.6 ^b^	25.7 ± 3.7 ^b^	0.0486
C-peptide (nmol/L)	1.5 ± 0.2 ^b^	1.5 ± 0.1 ^b^	2.0 ± 0.3 ^a^	1.3 ± 0.1 ^b^	1.1 ± 0.1 ^b^	0.0126
HOMA-IR	24.5 ± 3.9 ^b^	34.8 ± 5.0 ^ab^	42.8 ± 7.0 ^a^	26.3 ± 5.0 ^b^	20.3 ± 3.6 ^b^	0.0179
Estradiol (ng/L)	26.4 ± 4.2	23.7 ± 3.9	23.3 ± 3.3	24.0 ± 3.4	20.9 ± 3.3	0.8780

Values are presented as means ± SEM (*n* = 10 per group). Sham, sham-operation; HFD, high-fat diet; OVX, ovariectomized; Low, 30 mg/kg Soy-Hop; Medium, 100 mg/kg Soy-Hop; High, 300 mg/kg Soy-Hop; LDL-cholesterol, low density lipoprotein-cholesterol; HDL-cholesterol, high density lipoprotein-cholesterol; TC, total cholesterol; TG, triglycerides; FBG, fasting blood glucose; HOMA-IR, homeostasis model assessment of insulin resistance. Different letters in the same row are significantly different at *p* < 0.05 as analyzed by Duncan’s multiple comparison test.

**Table 2:** Effects of Soy-Hop on biochemical and mRNA expressions related to bone loss in OVX/HFD rats. Rats were orally administered with Soy-Hop (30, 100, and 300 mg/kg) for 8 weeks.

Variables	Sham/HFD	OVX/HFD				*p*-Values
		Control	Low	Medium	High	
Osteocalcin (pg/mL)	2824.8 ± 114.1 ^b^	3749.0 ± 372.8 ^a^	2388.4 ± 124.3 ^bc^	2938.7 ± 266.3 ^b^	1919.9 ± 87.1 ^c^	<0.0001
ALP (ng/mL)	446.2 ± 87.8 ^ab^	717.2 ± 208.2 ^a^	377.9 ± 103.1 ^ab^	266.6 ± 64.2 ^b^	198.8 ± 107.9 ^b^	0.0474
CTX (ng/mL)	11.4 ± 0.4 ^c^	15.9 ± 0.8 ^a^	14.7 ± 0.6 ^ab^	15.4 ± 1.1 ^a^	12.3 ± 1.6 ^bc^	0.0122
NTX (nM BCE)	59.8 ± 3.2	73.2 ± 4.3	71.7 ± 6.1	62.6 ± 3.7	68.1 ± 5.9	0.2493
RANKL	1.0 ± 0.2 ^b^	1.5 ± 0.2 ^a^	1.3 ± 0.1 ^ab^	1.2 ± 0.1 ^ab^	1.2 ± 0.1 ^ab^	0.0671
OPG	1.0 ± 0.5	0.9 ± 0.3	1.0 ± 0.4	1.4 ± 0.4	1.6 ± 0.5	0.6675
RANKL/OPG	1.0 ± 0.2 ^ab^	1.4 ± 0.4 ^a^	0.6 ± 0.3 ^b^	0.5 ± 0.2 ^b^	0.4 ± 0.2 ^b^	0.0610

Values are presented as means ± SEM (*n* = 10 per group). Sham, sham-operation; HFD, high-fat diet; OVX, ovariectomized; Low, 30 mg/kg Soy-Hop; Medium, 100 mg/kg Soy-Hop; High, 300 mg/kg Soy-Hop; ALP, alkaline phosphatase; CTX, collagen type 1 cross-linked C telopeptide; NTX, collagen type 1 cross-linked N telopeptide; RANKL, receptor activator of nuclear factor kB ligand; OPG, osteoprotegerin. Different letters in the same row are significantly different at *p* < 0.05 as analyzed by Duncan’s multiple comparison test.

**Table 3:** Composition of the high-fat diet.

**Formulation**	**g %**	**kcal %**
Protein	24	20
Carbohydrate	41	35
Fat	24	45
kcal/kg	4776	
**Ingredients**	**g**	**kcal**
Casein (from milk)	238.8	800
Corn starch	185.1	620
Sucrose	59.7	200
Dextrose	157.6	528
Cellulose	59.7	0
Soybean oil	29.9	225
Lard	208.9	1575
Mineral mixture	41.8	0
Vitamin mixture	11.9	40
*tert*-Butylhydroquinone	0.02	0
l-Cysteine	3.6	12
Choline bitartrate	3.0	0
Total	1000.02	4000

**Table 4:** Primer sequences for real-time qPCR.

Name	GenBank No.	Sequence (5′-3′)	Amplicon Size (bp)
RANKL	NM057149	F: 5′-GCA GCA TCG CTC TGT TCC TGT A-3′	164
		R: 5′-GCA TGA GRC AGG TAG TGC TTC TGT G-3′	
OPG	NM012870	F: 5′-GGC AGG GCA TAC TTC CTG TT-3′	109
		R: 5′-GCC ACT TGT TCA TTG TGG TCC-3′	
β-actin	NM031144	F: 5′-CTC TGT GTG GAT TGG TGG CT-3′	150
		R: 5′-GGG TGT AAA ACG CAG CTC AG-3′	
